# Probing Effect of Papirindustriens Forskningsinstitut (PFI) Refining on Aggregation Structure of Cellulose: Crystal Packing and Hydrogen-Bonding Network

**DOI:** 10.3390/polym12122912

**Published:** 2020-12-04

**Authors:** Kunpeng Li, Lihong Zhao, Beihai He

**Affiliations:** State Key Laboratory of Pulp and Paper Engineering, South China University of Technology, 381 Wushan Rd., Tianhe District, Guangzhou 510640, China; felikp@mail.scut.edu.cn (K.L.); ppebhhe@scut.edu.cn (B.H.)

**Keywords:** natural cellulosic fibers, mechanical refining, Crystallinity, Crystallite size, Hydrogen bonds

## Abstract

Supramolecular structure is the critical factor that affects the properties of cellulosic fibers. This article studied the action of Papirindustriens forskningsinstitut (PFI) refining on the molecular aggregation and hydrogen bonding network, and tried to explore the relationship between the crystal packing and hydrogen-bonding network in cellulosic fibers. The results showed that the polymorph, H-bonding distance, and H-bonding energy of various H-bonds remained almost unchanged, while the crystalline index, crystallite size, and content of various H-bonds changed with refining. Therein, the content of the inter-molecular O(6)H⋯O(3′) H-bonds was significantly correlated with the crystalline index that was obtained in intensities of the XRD peaks. The Pearson correlation coefficient between them was 0.888 (*p* < 0.05) for softwood fibers and 0.889 (*p* < 0.05) for hardwood fibers, respectively. It can be concluded that the variations of accessibility, swelling, and fibrillation were closely related to the supramolecular structure and the intermolecular H-bonds play an important role in the crystal packing of cellulose.

## 1. Introduction

Mechanical, chemical, and biological treatments are usually adopted to obtain the desired physical and mechanical properties and for further utilization of the natural cellulosic fibers. Therein, Papirindustriens forskningsinstitut (PFI) refining is the most common laboratory refiner in order to improve the inter-fiber bonding capacity [1] and accessibility [2,3], and manufacture the micro-fibrillated or nano-fibrillated cellulose [4,5,6]. The main effects of PFI refining on fibers are: (i) internal fibrillation—delaminating, loosening, and swelling of fiber wall; (ii) external fibrillation—peeling of fibrils from the fiber surface while remaining attached; (iii) fiber shortening—fiber cutting due to shearing action, and (iv) fines formation—primary fines come from ray cells and parenchyma cells and microfibrils that are caused by fiber shortening or external fibrillation. Moreover, the PFI refining could be adopted to obtain a higher internal fibrillation than other laboratory refiners [1]. These effects of the PFI refining on cellulosic fibers will improve the accessibility, the swelling, the flexibility, and the inter-fiber bonding capacity of fibers. It is assumed that these changes in the macro-level structure and properties are related to the changes of crystallinity and H-bonding network during refining [7,8]. 

There are some different conclusions regarding to the effects of PFI refining on crystallinity of cellulose, not like that of ball milling, which effectively reduces the crystallinity as the treatment progresses. Although some researchers considered that the crystallinity decreased during PFI refining for corn stover [9] or softwood pulp [10], some researchers reported that the crystallinity increased first and then decreased with the degree of refining for hardwood bleached kraft pulp fibers [11] or for unbleached eucalyptus fibers [12]. Moreover, Zhao et al. [7] reported that the crystallinity initially fluctuated upward and then downward, followed by a repeating fluctuation with PFI refining for tobacco stem mechanical pulp. None of the previous studies identified a clear trend, but the results were that the crystallinity varied during PFI refinement. Based on the large proportion of H-bonds in crystalline region of cellulose, it can be assumed that the H-bonding network might change during PFI refining.

Fourier transformation infrared spectroscopy (FT-IR), which is sensitive to molecular structure, can be as an indirect measurement for studying H-bonding network. FT-IR deconvolution of hydroxyl groups band was adopted in order to study the changes of the energy of H-bonds and the H-bonding distance during delignification [13] or the deconstruction of cellulose [14]. Moreover, it was used to study the differences of the content of H-bonds not only between dislocation region and normal region [15], but also among plant species [16,17]. Regarding the changes of H-bonding network during PFI refining, Yuan et al. [18] reported that the content of H-bonds and crystallinity index (CI) of eucalyptus fibers changed with the increment of beating time. Subsequently, Zhao et al. [8] found that the H-bonding energy for O(6)H⋯O(3′) inter-molecular H-bonds decreased by 12.9% and evolutions of the content of certain H-bonds differed at various refining stages. 

There is still a lack of literature on the effects of PFI refinement on the H-bonding network. Particularly, the relationship between the content of H-bonds and crystallinity is still worthy of further study, which will deepen the understanding of the molecular aggregation and distribution of H-bonds, providing a molecular-scale explanation for the variations in accessibility, swelling, flexibility, and fibrillation of cellulosic fibers during PFI refinement. Therefore, this work explored the effects of PFI refinement on the CI, crystallite size, energy of H-bonds, H-bonding distance, and content of H-bonds for softwood pulp fibers and hardwood pulp fibers. Attempts were made in order to establish the correlations between the CI and content of various H-bonds. 

## 2. Methods

### 2.1. PFI Refining

Commercial bleached softwood and hardwood kraft pulp fibers were obtained from Zhongzhirenhe Company (Jinan, Shandong Province, China). Two pulp were treated with a PFI refiner (Hamjern Maskin A/S, Hamar, Norway) according to Tappi T248 wd-97, with a pulp consistency of 10%, the gap between the opposite refining plates was 0.2 mm, and forces per bar length were 3.33 N/mm and 1.77 N/mm for softwood fibers and hardwood fibers, respectively. The drainability was characterized by the Schopper Riegler number (SR-number) with the standards ISO 5267-1. Two pulp fibers were separately refined in a PFI refiner at 0, 4000, 8000, 12,000, and 16,000 revolutions and at 0, 4000, 8000, 12,000, and 16,000 revolutions, respectively. Corresponding various pulps with an increasing beating degree, such as 16, 20, 40, 63, and 79° SR, were obtained for softwood fibers and 0, 26, 40, 57, and 70° SR were obtained for hardwood fibers.

### 2.2. Morphological Analysis of Fibers and Mechanical Properties of Sheets

The fines content, weighted mean length, and macro-fibrillation index of each sample were determined with a MORFI LB analyzer (Techpap SAS, Grenoble, France). The tensile strength of dry sheets and Wet-web strength were tested according to TAPPI T494 and Chinese standard GBT 12914-2008, respectively. The zero-span tensile strength was tested with the Z-Span 2400 apparatus (Pulmac International Co. Ltd., Montpelier, VT, USA), according to the standard ISO 15361.

### 2.3. Water Retention Value (WRV)

The *WRV* was determined with 0.5 g samples (o.d.) according to ISO 23714.

(1) $$WRV\left(\%\right)=\frac{\left(m_{1}-m_{2}\right)}{m_{2}}\times 100\%$$

where *m*_1_ is the weight of the sample after centrifugation and *m*_2_ is the weight of the oven-dried sample.

### 2.4. XRD Analysis

The crystallinity of pretreated fibers was determined by a Shimadzu XRD 7000S (Shimadzu, Kyoto, Japan), while using Cu-Ka radiation. The samples were scanned at 40 kV and 30 mA in a 2θ range of 4–40° at 1°/min. It is well known that two polymorphs, cellulose Iα and Iβ, are present in native cellulose; for clarity, all of the reflections indexed here are on the Iβ lattice. Before analysis, the experimental data is nine point smoothed with Savitzky–Golay algorithm. The two types of CI values were obtained from peak intensity method (*CI − XI*) and peak fitting (CI-XA), respectively. The *CI − XI* was calculated as in Equation (2) [19].

(2) $$CI-XI=\frac{\left(I_{200}-I_{am}\right)}{I_{200}}\times 100\%$$

where *I200* is the maximum diffraction intensity value of the (200) lattice plane between the scattering angles of 2θ = 22° and 23°, and *I_am_* is the minimum diffraction intensity of amorphous fraction between 2θ = 18° and 19°. 

All of the XRD diffractograms were smoothed with the Savitzky–Golay algorithm and baseline-corrected with linear fit function. Subsequently, the baseline-corrected diffractograms were deconvoluted with the Pearson VII function. The value of the CI-XA equals the area percentage occupied by crystalline peaks over the total area of the diffraction pattern [20]. In addition, the crystallite size was calculated by the widely used Scherrer Equation (3) [21]:

(3) $$L=\frac{k\times \lambda }{\left(\beta \times \cos\theta \right)}\;$$

where *L* is the size that is perpendicular to the lattice plane, *k* is a constant that depends on the crystal shape (0.89), λ is the wavelength of the incident beam in the diffraction experiment (0.154 nm), *β* is the full width at half maximum of the diffraction peak, and *θ* is the Bragg’s angle. In general, the values of the calculated crystallite sizes might be lower than that of the real crystallite sizes, because the peak broadening from the instrumental factors was not considered [22].

### 2.5. FT-IR Analysis

THE FT-IR spectra were recorded on solid samples in KBr pellets by means of an FT-IR Bruker Vertex 70 spectrometer (Karlsruhe, Germany). Unrefined and refined fibers were smashed into powder between 40 and 60 mesh. Subsequently, the powder sample of each species (5 mg) was dispersed in a matrix of highly pure KBr (500 mg). The spectral was recorded by averaging 64 recordings and at a resolution of 4 cm^−1^ with transmission mode from 4000 cm^−1^ to 400 cm^−1^. The results were made on the average spectrum that was obtained from five recordings.

The energy of the H-bonding E_H_ for several OH stretching bands was calculated while using Equation (4) [13].

(4) $$E_{H}=\frac{\nu_{o}-\nu }{\nu_{o}}$$

ν_o_ is the wavenumber corresponding to free OH groups (3650 cm^−1^), ν is the frequency (cm^−1^) of the bonded OH groups, and k is a constant (1/k = 2.625 × 102 kJ).

The H-bonding distance R is obtained while using the Pimentel and Sederholm equation [23], as follows.

(5) $$△\nu \left({\mathrm{cm}}^{-1}\right)=4430\times \left(2.84-R\right)$$

△ν = ν_o_ − ν, ν_o_ is the monomeric OH stretching frequency, which is taken to be 3600 cm^−1^, ν is the stretching frequency (cm^−1^) observed in the infrared spectrum of the sample, and R is the H-bonding distance (Å).

The spectral was normalized at 1016 cm^−1^ and the derivative curve was obtained by applying the Savitzky–Golay algorithm. The wavenumbers of H-bonding components are taken from second-derivative spectra according to regions that are assigned to various H-bonds. Nonlinear fitting of the FT-IR spectral region from 3000 cm^−1^ to 3700 cm^−1^ was carried out with Gaussian function in Peakfit 4.12 software (SeaSolve Software Inc., Framingham, MA, USA). The Pearson correlation coefficients were evaluated between the CI and content of various H-bonds, with a statistically significant *p*-value of <0.05, while using Spss 25.0 statistics software (IBM Corp., Armonk, NY, USA).

## 3. Results and Discussions

### 3.1. The Morphological Analysis, Swelling, and Mechanical Properties

**Figure 1:**
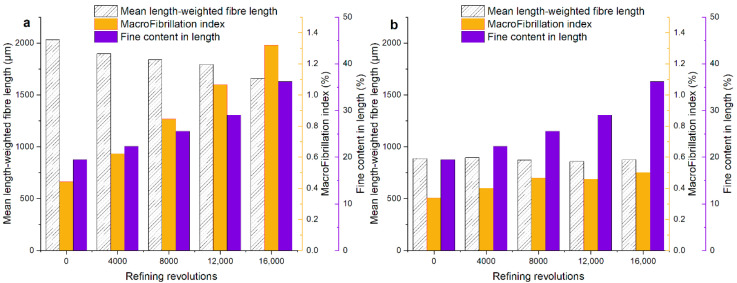
The morphological analysis for the unrefined and refined samples: (**a**) softwood fibers and (**b**) hardwood fibers.

For softwood fibers and hardwood fibers, the macro-fibrillation index and the fiber length had a downward trend, and the fines content has an upward trend for the softwood fibers during PFI refining, as can be seen in Figure 1. The macro-fibrillation index and fiber length of softwood fibers changed significantly. However, two parameters of hardwood fibers had no significant variations relative to softwood fibers. It might be because of the lower refining pressure that was applied in hardwood fibers. The macro-fibrillation index characterizes external macro-fibrillation. The results of fiber morphology analysis showed that external fibrillation increased with the refining process. It is consistent with the increasing specific surface area (as shown in Table S1 in Online Resource). 

**Figure 2:**
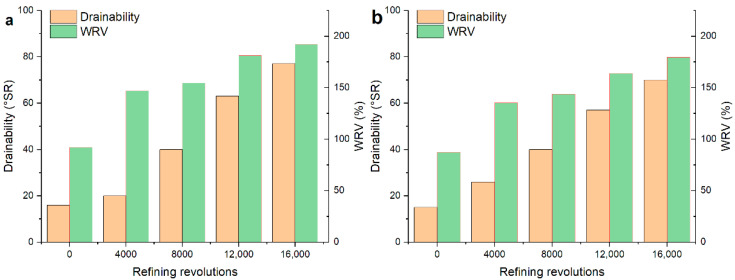
The beating degree and Water Retention Value (WRV) for the unrefined and refined samples: (**a**) softwood fibers and (**b**) hardwood fibers.

The beating degree and the WRV had a significant increment with increasing PFI refining revolutions, as can be seen in Figure 2. It is caused by the higher fibrillation that produces the new surface and more pores (as shown in Table S1 in Online Resource). The increase in swelling characterized by WRV makes the fibers more compliant in transverse [1], which contributes to improving the inter-fiber bonding strength of the fiber network. The results of fiber morphology analysis and water retention value showing that the fibrillation and the separation between fibrils increase with the refining process. 

**Figure 3:**
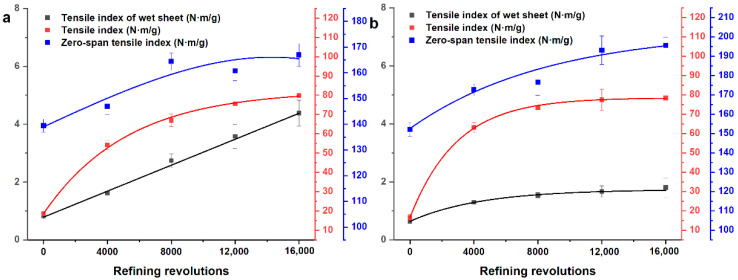
The rheological analysis of wet sheets, the tensile strength of dry sheets, and the fiber strength for unrefined and refined samples: (**a**) softwood fibers and (**b**) hardwood fibers.

In Figure 3, the tensile index, zero-span tensile index, and wet-web strength represented as a function of the refining revolutions. The increment of tensile strength is mainly caused by the increasing fibrillation and fines during PFI refining, as mentioned above. The wet–web strength of softwood fiber had a more significant increase when compared to that of hardwood fiber. It is for that fiber length plays an essential role in wet–web strength. The zero-span tensile index was usually used in characterizing the tensile strength of fibers. The results of the zero-span tensile index showed that PFI refining did not reduce fiber strength, which is consistent with the report of Kouko et al. [24]. The above results showed that PFI refining improved the extent of fibrillation (especially the internal fibrillation), the swelling of fibers, and the tensile strength of sheets without a decrease of the tensile strength of fibers.

### 3.2. Effect of PFI Refining on Crystalline Index and Crystallite Size

**Figure 4:**
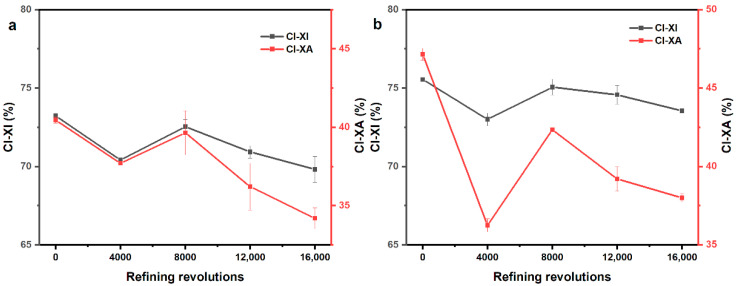
The CI-XI (crystallinity index obtained by comparing intensity of X-ray diffractometer (XRD) peaks) and CI-XA (crystallinity index obtained by comparing area of XRD peaks) for refined and unrefined samples: (**a**) softwood fiber and (**b**) hardwood fiber.

The cellulose polymorph of these two fibers had no change during PFI treatment, which illustrates that the PFI refining is a relatively mild mechanical treatment, as the results of XRD in Online Resource (Figures S1 and S2) show. In general, the CI-XI and the CI-XA of softwood fibers showed a reduction of 4.66% and 15.47%, respectively, and those of hardwood fibers decreased by 2.65% and 19.4%, respectively, as can be seen in Figure 4 and Table 1. The value of the CI-XI was remarkably higher than the CI-XA, which might be caused by the over-fitting of amorphous contribution in the analysis of CI-XA. However, the CI-XI and the CI-XA were correlated significantly for both softwood fibers and hardwood fibers, with a correlation coefficient of 0.919 (*p* < 0.05) for softwood fibers and 0.925 (*p* < 0.05) for hardwood fibers, respectively, which is consistent with the report of Ahvenainen et al. [25]. 

The trends of CI (CI-XI and CI-XA) for softwood fibers and hardwood fibers were all similar, decreasing first and then increasing, along with a decline, as shown in Figure 4. The fluctuation of the CI was probably because of the mechanical action of PFI refining on the different regions of cellulose. In the first refining stage, the decrease of the CI showed that mechanical treatment may mainly act in the crystalline region. Subsequently, the growth of the CI could be interpreted as greater force acting on the amorphous region in which cocrystallization might occur upon drying under the mechanical and thermal action of PFI refining. The subsequent decrease might be the continuous destruction of crystalline region.

**Figure 5:**
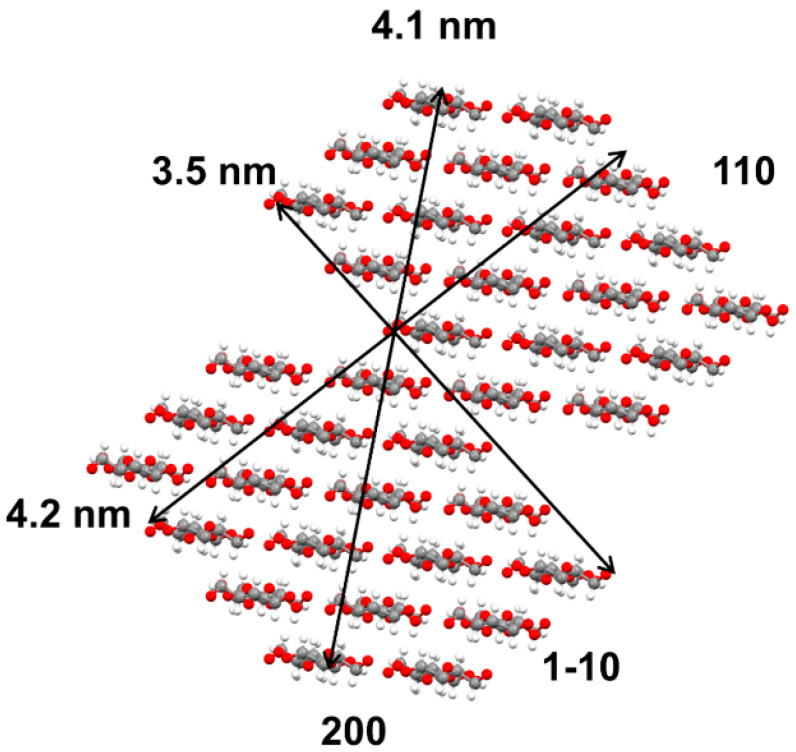
Cross-section model of cellulose crystallite for softwood fibers and hardwood fibers.

Recently, researches indicated that plant cell walls commonly contained microfibrils with 18 cellulose chains [26]. Frequently adopted arrangements of the 18 cellulose chains are 234432, 34443, and 333333 [27]. Therein, 234432 arrangement was found to be more probable based on calculation [28]. Thus, according to the crystallite sizes (as Table 1 shows) and the unit cell parameters of cellulose Iβ, there was most likely to be an average of two-fibrils with an arrangement of 234,432 packed side-by-side for both softwood fibers and hardwood fibers (as seen in Figure 5, drawn with mercury 4.1.2 software). The result is consistent with the report of Haigler & Roberts [26], which shows that there might be two microfibrils associated along part of their length. 

It is known that both the ($1\bar{1}0$) and the (110) lattice plane are hydrophilic, and the (200)lattice plane is hydrophobic in cellulose Iβ (as Figure 5 shows). In addition, it is noticeable that the (110) planes possess the highest theoretical roughness and accessibility to the hydrophilic groups in cellulose Iβ [28]. Throughout the refining of two pulp fibers, the sizes perpendicular to (110) plane were more volatile than the ($1\bar{1}0$) plane and (200) plane, which is consistent with the report of Duchemin et al., as shown in Table 1a,b [29]. It could be interpreted to be that water molecules were easier to permeate through the spaces between the hydrophilic (110) planes in paracrystalline region (hydration), and then it made these planes suffer more mechanical actions, so there was more adhesion (cocrystallization) and cleavage of cellulose chains created for (110) plane during PFI refining. 

However, the crystallite size that was perpendicular to (200) lattice plane of two fibers had an upward trend during PFI refining. It could be explained in terms of coalescence of chains or some crystallographic stacking of adjacent microfibrils, where the (200) planes were aligned upon drying [30]. Additionally, it was probably due to the weaker cleavage effect on (200) lattice planes that was hard for water molecules to penetrate. 

### 3.3. Effect of PFI Refining on H-Bonding Energies, H-Bonding Distances, and Content of H-Bonds

A mixture of inter-molecular and intra-molecular H-bonds is considered to be the cause for the broadening of OH bands in the FT-IR spectra. Therefore, taking derivative of the OH bands can improve the resolution of various H-bonds. Normally, 3580–3550 cm^−1^ is for free OH(2) and OH(6), 3455–3410 cm^−1^ is for O(2)H⋯O(6) intra-molecular H-bonds, 3375–3340 cm^−1^ is for O(3)H⋯O(5) intra-molecular H-bonds, and 3310–3230 cm^−1^ is for O(6)H⋯O(3′) inter-molecular H-bonds in cellulose I [31,32].

**Figure 6:**
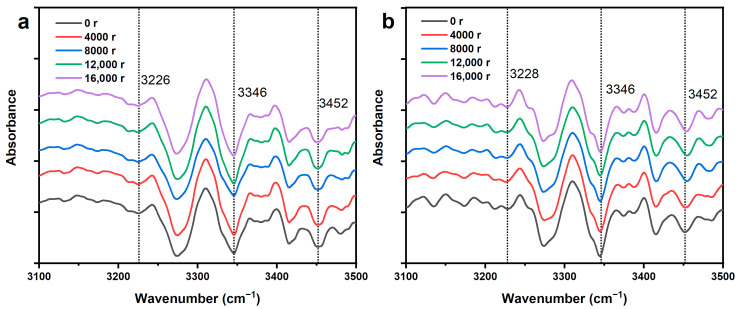
The second derivative of the Fourier transformation infrared spectroscopy (FT-IR) spectra for the refined and unrefined samples: (**a**) softwood fiber and (**b**) hardwood fiber.

The peak position assigned to O(6)H⋯O(3′) inter-molecular H-bonds (around 3227 cm^−1^), O(3)H⋯O(5) intra-molecular H-bonds (around 3346 cm^−1^), and O(2)H⋯O(6) intra-molecular H-bonds (around 3452 cm^−1^) of softwood fibers and hardwood fibers almost kept constant in the whole refining process, as shown in Figure 6a,b. According to the formulae of H-bonding energy and H-bonding distance (Equations (4) and (5)), the H-bonding energies and H-bonding distances of the three H-bonds remained unchanged, as the peak positions of three H-bonds almost did not exhibit red shift or blue shift. The results showed that PFI refining did not change the H-bonding energies and H-bonding distances, just as it did not change the cellulose polymorph in the above results.

**Figure 7:**
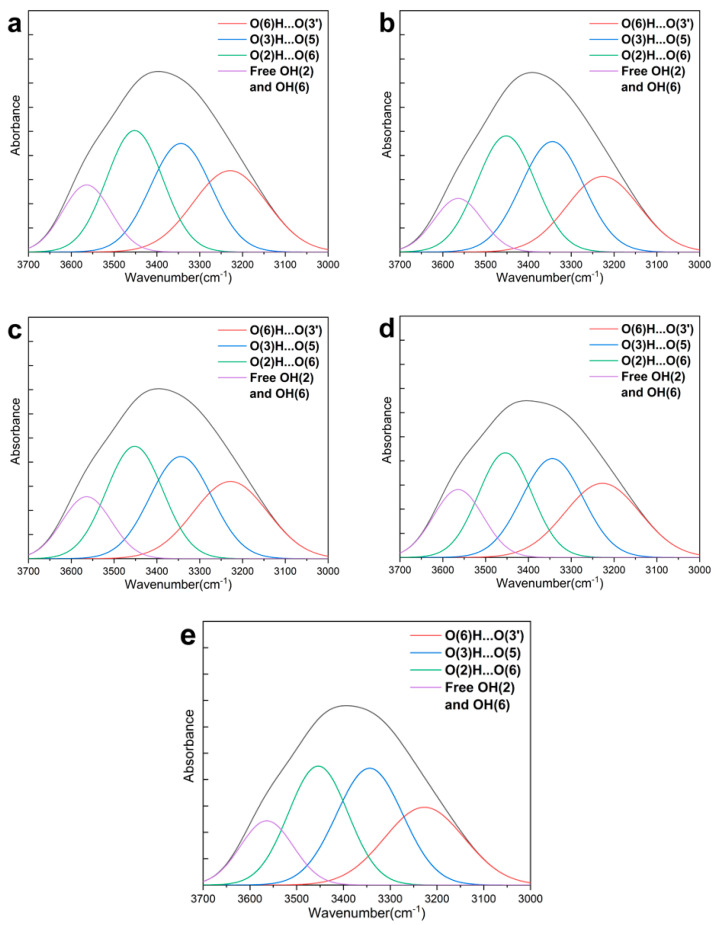
The deconvoluted FT-IR spectra of softwood fibers at various revolutions: (**a**) 0 r, (**b**) 4000 r, (**c**) 8000 r, (**d**) 12,000 r, and (**e**) 16,000 r.

Because the recorded infrared spectrum is the convolution of the real spectrum and the linear function of instrument. The band of hydroxyl groups (3000–3700 cm^−1^) was deconvoluted into four predominant bands in the present study based on the peak positions above. Subsequently, the relative content of various H-bonds was calculated based on area proportion of the band, respectively. Finally, the data and curves obtained using nonlinear fitting for softwood fibers is shown in Table 2a and Figure 7, and the results of hardwood fibers can be seen in Table 2b and Figure S3.

For the content of O(6)H⋯O(3′) inter-molecular H-bonds of softwood fiber, the trend can be divided into two stages, as shown in Table 2a. It decreased by 7.72% from 28.23% to 26.05% as refining from 0r to 4000 r and then increased by 9.14% from 4000 r to 8000 r, followed by a decrease of 17.13%, with the revolutions increasing from 8000 r to 16,000 r. Regarding the O(6)H⋯O(3′) H-bonds of hardwood fiber, the trend was almost similar to that of softwood fibers, except for a small increase of 0.45% from 8000 r to 12,000 r (in Table 2b). On the whole, the amount of the O(6)H⋯O(3′) H-bonds for softwood fibers and hardwood fibers decreased by 16.54% and 5.91% altogether, respectively. The decrease might be due to the cleavage among cellulose molecular chains under the mechanical action of PFI refining, which was closely related to internal and external fibrillation (as seen in Section 3.1). On the other hand, the increase illustrates that there was not only the breakage, but also the formation of O(6)H⋯O(3′) H-bonds. Both the fewer inter-molecular H-bonds and the lower CI (as Section 3.2 shows) will further improve the flexibility of cellulosic fibers, due to the higher mobility of molecular chains, the accessibility, and the WRV of fibers (as seen in Section 3.1). 

As to the O(3)H⋯O(5) intra-molecular H-bonds of softwood fiber, the content had a growth of 10.09% from 28.44% to 31.31% in the stage of refining from 0 r to 4000 r, and then had a slight decrease of 1.44% from 4000 r to 8000 r, followed by an increase of 13.16% until the end of refining. Overall, the trend was similar to that of hardwood fibers, which first increased and then had a decrease, followed by a growth. During the whole refining, the amount of O(3)H⋯O(5) H-bonds for softwood fibers and hardwood fibers showed an upward tendency and increased by 22.78% and 8.8%, respectively. The increase might be because the stable O(3)H⋯O(5) H-bonds might have not only less breakage under the action of water as destructor to the crystallites [30], but also creation in the amorphous region upon drying. Because hydrogen bond–covalent bond synergies play an important role in the cellulose stiffness axial modulus, more O(3)H⋯O(5) H-bonds will enhance the cellulose stiffness [33,34].

In the case of the O(2)H⋯O(6) intra-molecular H-bonds for softwood fiber, the trend of content can be described as two phases. It had an increase of 3.5% from 31.4% to 32.5% in the stage of refining from 0 r to 4000 r and it then decreased by 21.57% from 4000 r to 12000 r, along with a growth of 15.26% in the last refining stage. For the content of the O(2)H⋯O(6) H-bonds of hardwood fiber, it increased firstly and then had a decrease that was also followed by an increase. For both fibers, the content of O(2)H⋯O(6) H-bonds decreased, except for the stage of refining from 0 r to 4000 r. It might be due to the fact that the O(2)H⋯O(6) H-bonds, which are much more accessible to water, are less stable than O(3)H⋯O(5) H-bonds.

As to the free OH(2) and OH(6) of softwood fiber, the relative content showed a slight increase trend in general. The enhancement of the free OH(2) and OH(6) was understandable, because there was the breakage of H-bonds among cellulosic molecules, which resulted in more extensive delamination and higher specific surface area (as shown in Figure S4 and Table S1 in Online Resource). This made cellulose more accessible to water and chemicals. However, for the free OH(2) and OH(6) of hardwood fiber, the number of the free OH(2) and OH(6) decreased by 21.65% from 14.32% to 11.22%, with revolutions increasing from 0 r to 4000 r, followed by an increase of 45.37% in the stage of refining from 4000 r to 12,000 r, along with an decrease of 17.11%. It could be explained by the instability of free OH groups, which makes it easy to form new H-bonds, such as O(2)H⋯O(6) or O(6)H⋯O(3′)H-bonds. Thus although there is the formation of free OH(2) and OH(6) during fibrillation, the content of the free OH(2) and OH(6) might disease; meanwhile, the content of the O(6)H⋯O(3′) or the O(2)H⋯O(6) H-bonds might increase upon drying.

### 3.4. Correlation between CI and Content of Inter-Molecular H-Bonds during PFI Refining

**Figure 8:**
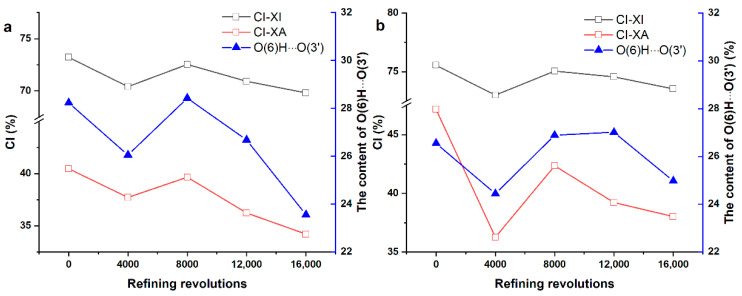
The trend of the CI-XI, the CI-XA, and the O(6)H⋯O(3′) H-bonds: (**a**) softwood fibers and (**b**) hardwood fibers.

**Figure 9:**
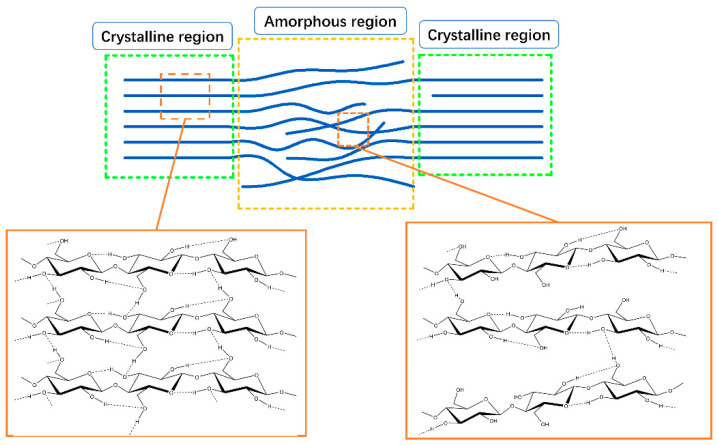
The illustration of a two-phase structure of cellulose.

Similar to the CI-XI, the content of O(6)H⋯O(3′) H-bonds decreased first and then had an increase, followed by a decline for the two fibers (as shown in Figure 8a,b). Besides, the Pearson correlation coefficients were calculated between the content of H-bonds and the CI-XI. It was 0.912 (*p* < 0.05) for softwood fibers and 0.889 (*p* < 0.05) for hardwood fibers, respectively. The similar trends in the content of the O(6)H⋯O(3′) H-bonds and the CI-XI might be because that the crystalline region has a higher content of O(6)H⋯O(3′) H-bonds when compared with the amorphous regions, as illustrated in Figure 9 [35].

The correlation between the CI-XI and the O(2)H⋯O(6) H-bonds or the free OH(2) and OH(6) was poor for the following reasons. The free OH groups are easier for forming the O(2)H⋯O(6) H-bonds as opposed to the O(6)H⋯O(3′) H-bonds in the amorphous region, because the distance between adjacent glucose units in a cellulosic molecular chain is closer than between the glucose units in the adjacent molecular chain (as shown in Figure 9). Accordingly, when compared with the content of O(6)H⋯O(3′) H-bonds, there was less difference in the content of the O(2)H⋯O(6) H-bonds or the free OH groups between the crystalline region and amorphous region, which sometimes made the content of the two bonds not change much in regards to the crystallinity. The O(3)H⋯O(5) H-bonds is stable, as mentioned above. It was presumed to change little as crystallinity changed during PFI refining. Hence, the correlation between O(3)H⋯O(5) H-bonds and the CI-XI was not discussed in the present study.

## 4. Conclusions 

PFI refining acts not only on the macro-level structure and mechanical properties, but also on the supramolecular structure, including crystallite size, crystallinity, and content of various H-bonds. The results indicated that the fibrillation and properties of fibers are related to the variation of the supramolecular structure during PFI refining. It is noticeable that there was a significantly positive correlation between the CI-XI and content of O(6)H⋯O(3)’ H-bonds during PFI refining for softwood fibers and hardwood fibers. Thus, the trend of the content of O(6)H⋯O(3)’ H-bonds might be generally speculated based on CI-XI while refining. Besides, the crystallite size, crystallinity, and content of various H-bonds did not change much in general. It is assumed that PFI refining is an excellent mechanical treatment of fibrillation with relatively little destruction on the supramolecular structure.

## Figures and Tables

**Table 1:** (**a**) The CI of softwood fibers through refining. (**b**) The CI of hardwood fibers through refining.

**(a)**					
**Revolutions**	**CI (%)**		**Crystallite Size (nm)**		
	**CI-XI ^a^**	**CI-XA ^b^**	**$1\bar{1}0$**	**(110)**	**(200)**
0	73.23	40.46	3.2	3.5	4.5
4000	70.42	37.71	3.1	3.4	4.6
8000	72.54	39.65	3.2	3.4	4.6
12,000	70.93	36.22	3.1	3.7	4.7
16,000	69.82	34.20	3.2	3.5	4.7
**(b)**					
**Revolutions**	**CI (%)**		**Crystallite Size (nm)**		
	**CI-XI ^a^**	**CI-XA ^b^**	**$1\bar{1}0$**	**(110)**	**(200)**
0	75.55	47.16	3.1	3.1	4.5
4000	73.01	36.26	3.1	3.3	4.7
8000	75.06	42.35	3.1	3.5	4.8
12,000	74.57	39.21	2.9	4.0	4.8
16,000	73.55	38.01	3.0	3.7	4.7

^a^ The CI-XI value was obtained from the ratio of the diffraction intensity. ^b^ The CI-XA value was obtained from the ratio of the peak areas.

**Table 2:** (**a**) The relative content of three H-bonds and the free OH for softwood fibers through refining. (**b**) The relative content of three H-bonds and the free OH for hardwood fibers through refining.

**(a)**					
**Revolutions**	**O(6)H** **⋯** **O(3ʹ)**	**O(3)H** **⋯** **O(5)**	**O(2)H** **⋯** **O(6)**	**Free OH(2) and OH(6)**	**r^2^**
0	28.23	28.44	31.40	11.93	0.9995
4000	26.05	31.31	32.50	10.14	0.9995
8000	28.43	30.86	25.87	14.84	0.9995
12,000	26.68	32.58	25.49	15.25	0.9996
16,000	23.56	34.92	29.38	12.14	0.9996
**(b)**					
**Revolutions**	**O(6)H** **⋯** **O(3ʹ)**	**O(3)H** **⋯** **O(5)**	**O(2)H** **⋯** **O(6)**	**Free OH(2) and OH(6)**	**r^2^**
0	26.55	29.09	30.04	14.32	0.9990
4000	24.44	33.06	31.28	11.22	0.9991
8000	26.89	29.81	28.99	14.31	0.9991
12,000	27.01	29.28	27.40	16.31	0.9992
16,000	24.98	31.65	29.85	13.52	0.9989

## Supplementary Materials

The following are available online at https://www.mdpi.com/2073-4360/12/12/2912/s1, Figure S1: XRD fitting results of softwood fibers: (a) 0, (b) 4000, (c) 8000, (d) 12,000, (e) 16,000 revolutions of PFI refining, Figure S2: XRD fitting results of hardwood fibers: (a) 0, (b) 4000, (c) 8000, (d) 12,000, (e) 16,000 revolutions of PFI refining, Figure S3: Nonlinear fitting curves of the band of hardwood fibers for hydroxyl groups: (a) 0, (b) 4000, (c) 8000, (d) 12,000, (e) 16,000 revolutions of PFI refining, Figure S4: The BET N2 adsorption/desorption isotherms at various refining revolutions of pulp fibers: (a) softwood; (b) hardwood, Table S1: Cumulative pore volume, average pore size and specific surface area of softwood pulp fiber through refining, Cumulative pore volume, average pore size and specific surface area of hardwood pulp fiber through refining.
