# Supplementary material for: Probing Effect of Papirindustriens Forskningsinstitut (PFI) Refining on Aggregation Structure of Cellulose: Crystal Packing and Hydrogen-Bonding Network
Source: Polymers (Basel). 2020 Dec 4;12(12):2912. doi: 10.3390/polym12122912 (PMC7761889; doi:10.3390/polym12122912)
Supplement: Supplementary file 1 [file polymers-12-02912-s001.zip › polymers-1023674-supplementary/Appendix.docx]

The CI and the content of hydrogen bonds of softwood fibers with PFI refining at 0, 4000, 8000, 12000, 16000, 20000, 24000, 28000 and 36000 revolutions.

The CI of softwood fibers through refining.

| **Revolutions** | **CI (%)** | |  | **Crystallite size (nm)** | | |
| --- | --- | --- | --- | --- | --- | --- |
|  | **CI-XI ^a^** | **CI-XA ^b^** |  | **10** | **110** | **200** |
| 0r | 73.23 | 40.46 |  | 3.2 | 3.5 | 4.5 |
| 4000r | 70.42 | 37.71 |  | 3.1 | 3.4 | 4.6 |
| 8000r | 72.54 | 39.65 |  | 3.2 | 3.4 | 4.6 |
| 12000r | 70.93 | 36.22 |  | 3.1 | 3.7 | 4.7 |
| 16000r | 69.82 | 34.20 |  | 3.2 | 3.5 | 4.7 |
| 20000r | 72.78 | 36.45 |  | 3.3 | 3.7 | 4.7 |
| 24000r | 73.05 | 35.33 |  | 3.2 | 3.6 | 4.8 |
| 28000r | 72.36 | 37.45 |  | 3.2 | 3.7 | 4.8 |
| 36000r | 70.34 | 32.24 |  | 3.3 | 3.5 | 4.8 |

The relative content of three H-bonds and the free OH for softwood fibers through refining.

| Revolutions | O(6)H⋯O(3ʹ) | O(3)H⋯O(5) | O(2)H⋯O(6) | Free OH(2) and OH(6) | r2 |
| --- | --- | --- | --- | --- | --- |
| 0r | 28.23 | 28.44 | 31.40 | 11.93 | 0.9995 |
| 4000r | 26.05 | 31.31 | 32.50 | 10.14 | 0.9995 |
| 8000r | 28.43 | 30.86 | 25.87 | 14.84 | 0.9995 |
| 12000r | 26.68 | 32.58 | 25.49 | 15.25 | 0.9996 |
| 16000r | 23.56 | 34.92 | 29.38 | 12.14 | 0.9996 |
| 20000r | 27.26 | 28.22 | 32.07 | 12.46 | 0.9986 |
| 24000r | 27.53 | 27.68 | 31.48 | 13.31 | 0.9988 |
| 28000r | 27.06 | 28.28 | 32.81 | 11.84 | 0.9987 |
| 36000r | 25.33 | 29.39 | 33.69 | 11.53 | 0.9989 |

The correlation coefficient between the CI-XI and the content of intermolecular hydrogen bonds is 0.888 (p<0.05) for softwood fibers with PFI refining.The else data of softwood fibers with PFI refining at 0, 4000, 8000, 12000, 16000, 20000, 24000, 28000 and 36000 revolutions.





The morphological analysis for the unrefined and refined softwood fibers.





The beating degree and WRV for the unrefined and refined samples





The rheological analysis of wet sheet, the tensile strength of sheet and the fiber strength for unrefined and refined softwood fibers.





The second derivative of the FT-IR spectra for the refined and unrefined softwood fibers.





The BET N2 adsorption/desorption isotherms at various refining revolutions of pulp fibers: (a) softwood; (b) hardwood.
