# Supplementary material for: Probing Effect of Papirindustriens Forskningsinstitut (PFI) Refining on Aggregation Structure of Cellulose: Crystal Packing and Hydrogen-Bonding Network
Source: Polymers (Basel). 2020 Dec 4;12(12):2912. doi: 10.3390/polym12122912 (PMC7761889; doi:10.3390/polym12122912)
Supplement: Supplementary file 1 [file polymers-12-02912-s001.zip › polymers-1023674-supplementary/Supplementary Data-polymers.docx]

**Supporting Information**

Probing effect of PFI refining on aggregation structure of cellulose: crystal packing and hydrogen-bonding network


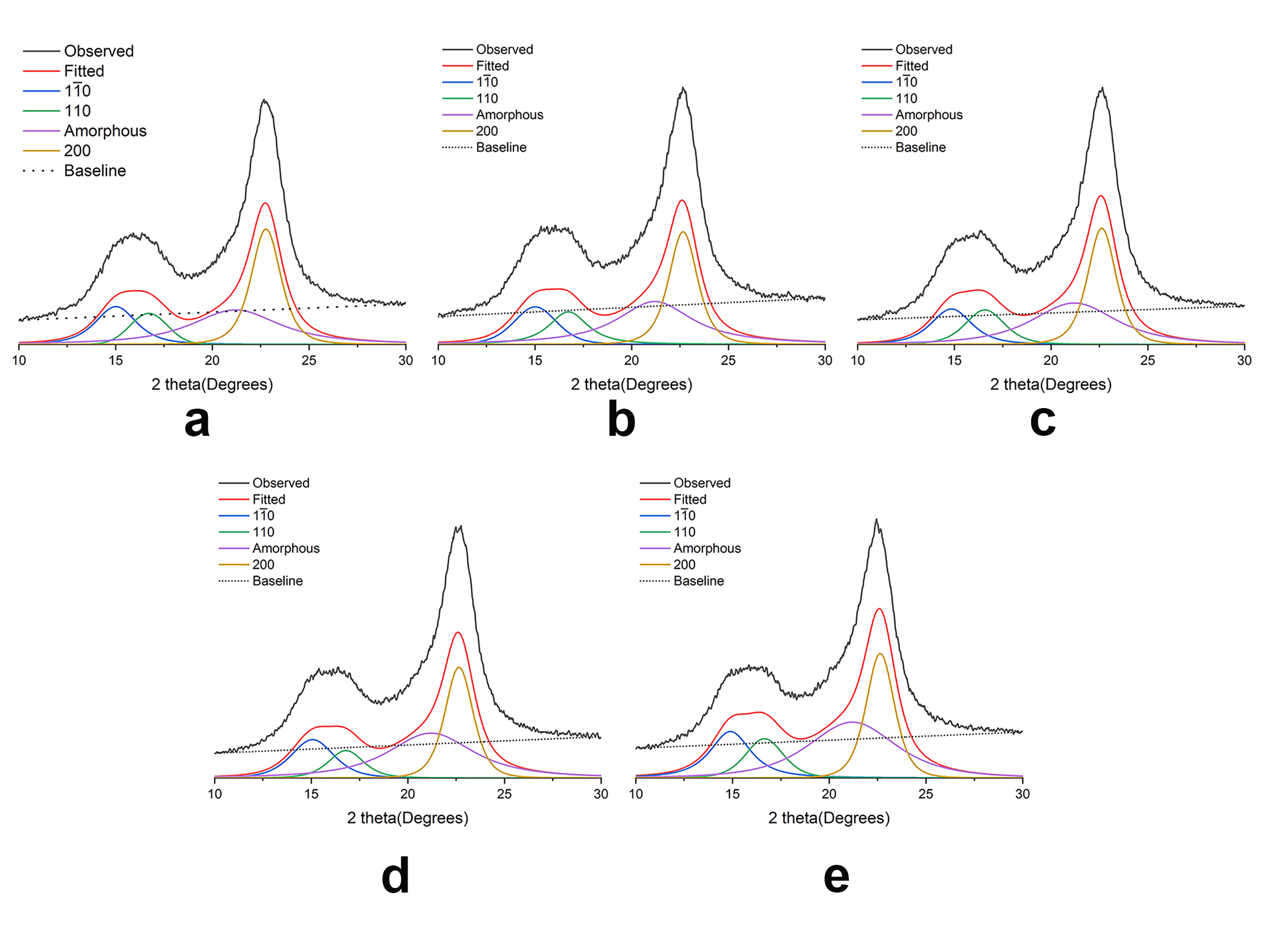


**Fig A1.** XRD fitting results of softwood fibers: a) 0, b) 4000, c) 8000, d) 12000, e) 16000 revolutions of PFI refining.


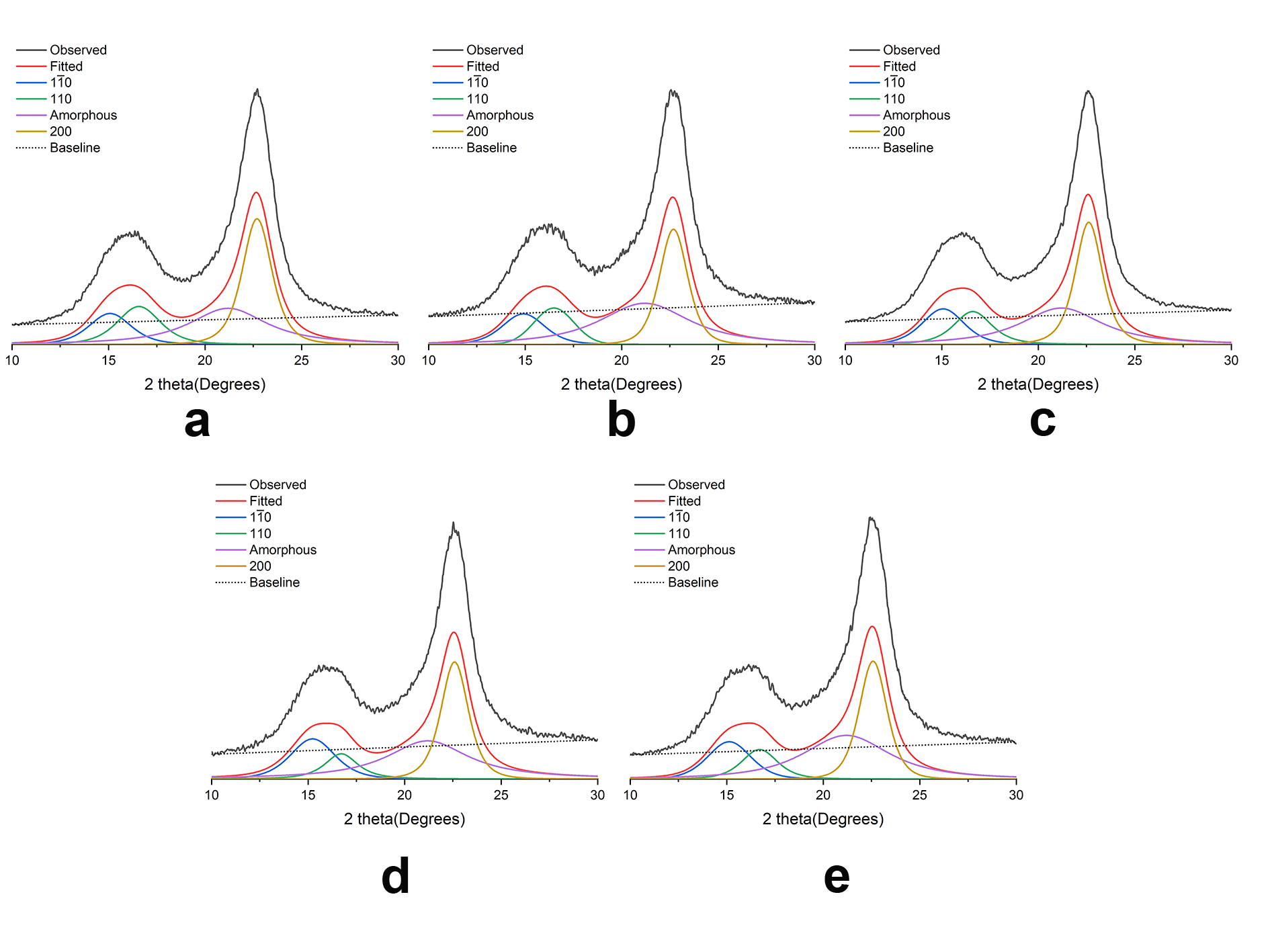


**Fig A2**. XRD fitting results of hardwood fibers: a) 0, b) 4000, c) 8000, d) 12000, e) 16000 revolutions of PFI refining.


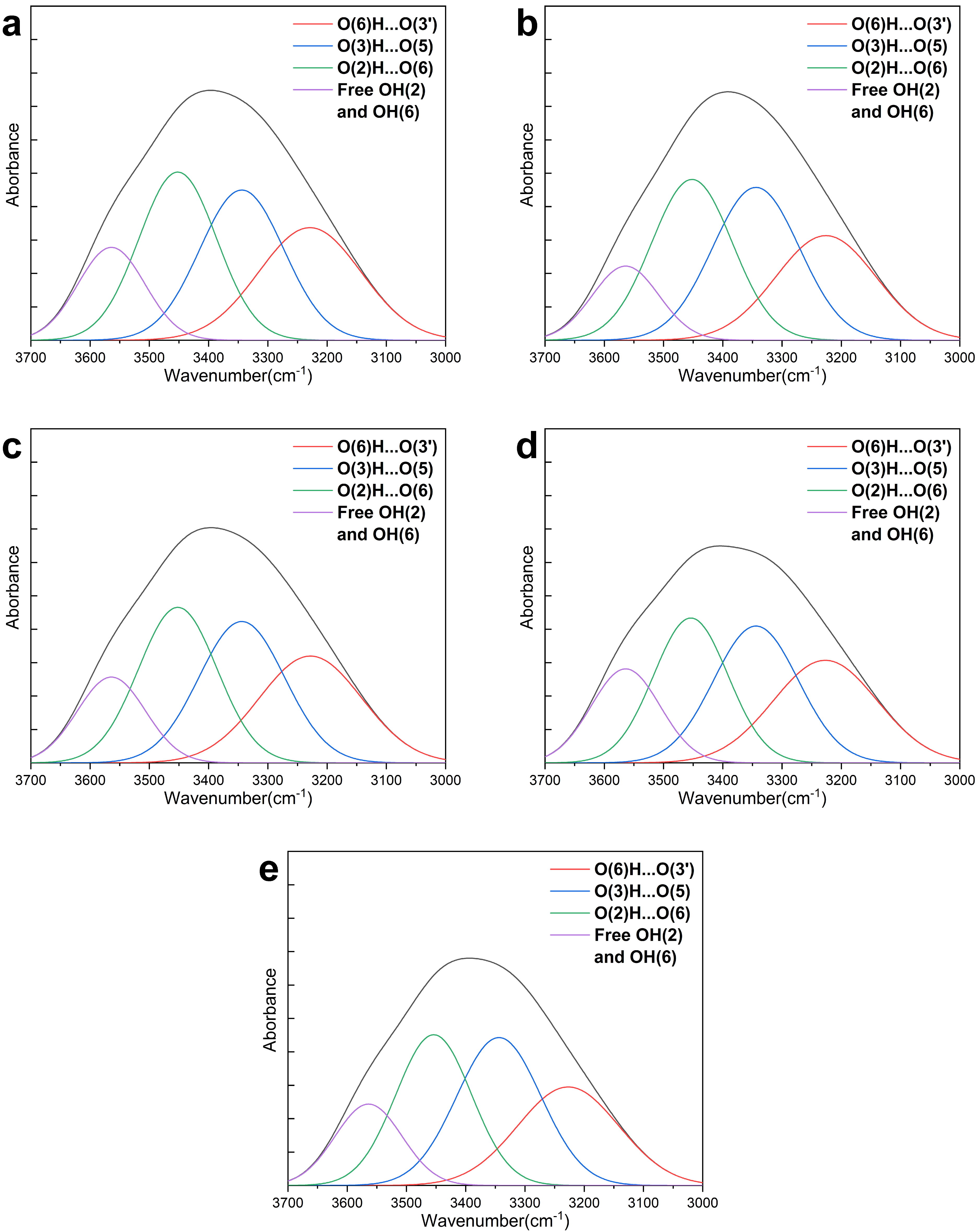


**Fig A3.** Nonlinear fitting curves of the band of hardwood fibers for hydroxyl groups: a) 0, b) 4000, c) 8000, d) 12000, e) 16000 revolutions of PFI refining.

Low-temperature nitrogen absorption analysis

Prior to testing, the fiber samples were dried at 60℃ for a whole night. A pore size distribution detector ASAP 2460 (USA, Micromeritics) was used to analyze the specific surface area, the cumulative pore volume and the average pore size High-purity N2 was used as an absorbate and the adsorption–desorption of N2 was determined at 77 K in a liquid nitrogen trap using a static volumetric method.







**Fig A4**. The BET N2 adsorption/desorption isotherms at various refining revolutions of pulp fibers: (a) softwood; (b) hardwood.

**Table A1a.** Cumulative pore volume, average pore size and specific surface area of softwood pulp fiber through refining.

| Refining Revolutions | Cumulative pore  volume (cm³/g) | Average pore  size (nm) | BET specific surface  Area (m²/g) |
| --- | --- | --- | --- |
| 0 | 0.002578 | 15 | 0.7429 |
| 4000 | 0.006074 | 20.4 | 1.2346 |
| 8000 | 0.005541 | 18.9 | 1.2416 |
| 12000 | 0.006452 | 17.1 | 1.6240 |
| 16000 | 0.006855 | 16.8 | 1.7485 |

**Table A1b.** Cumulative pore volume, average pore size and specific surface area of hardwood pulp fiber through refining.

| Refining Revolutions | Cumulative pore  volume (cm³/g) | Average pore  size (nm) | BET specific surface  Area (m²/g) |
| --- | --- | --- | --- |
| 0 | 0.002578 | 16.7 | 0.4242 |
| 4000 | 0.005040 | 18.9 | 1.1187 |
| 8000 | 0.004883 | 22.2 | 0.9022 |
| 12000 | 0.005223 | 18.8 | 1.1617 |
| 16000 | 0.006332 | 15.9 | 1.7244 |

All the unrefined and refined samples of two pulp fibers exhibited a type-IV N2 isotherm with a distinct hysteresis loop in Figure. S4. It suggested that slit pores presented in fibers. As shown in Table S1a, for the cumulative pore volume of softwood pulp, it had a substantial rise for about 136% from 0.002578 cm³/g to 0.005040 cm³/g with the revolution increasing from 0r to 4000r. And then it had a slight decline of 8.78% from 4000r to 8000r, followed by a sustained growth of 23.71% until the end of PFI refining. As to the of hardwood fiber, the cumulative pore volume had a similar trend to that of softwood fiber. Overall, both fibers had an upward tendency during the whole refining process. For two fibers, the rise of the cumulative pore volume was due to the formation of new pores through the fibrillation of cell wall, and the decrease in the stage of refining from 4000r to 8000r might be due to the destruction of the pores or the expansion of the macropores which was beyond the range of the nitrogen adsorption volumetric measurement.

As to the average pore size of softwood fiber, it increased firstly by 36% from 15 nm to 20.4 nm in the stage of refining from 0r to 4000r, then maintained a decrease of 17.65% in the following refining. In the case of hardwood fiber, it also increased firstly and then decreased. For two fibers, the increase in average pore size was caused by the expansion of pores. And the following decrease might be due to the formation of the mesopores and the micropores with smaller size.

As shown in Table S1, specific surface area of softwood fiber had a significant increase by about 135% from 0.7429 m²/g to 1.7485 m²/g with the increment of refining revolutions. And that of hardwood fiber also had an overall upward trend. The increase of the specific surface area was due to the new surface developed during PFI refining, which was consistent to the fact that fibrillation of fibers and formation of fines. The increment of specific surface area is dependent on fibrillation and fines formation during PFI refining [1]. More specifically, on the one hand the external fibrillation produces fines and pulls out microfibrils from fiber surface, on the other hand the internal fibrillation results in the formation of pores and more extensive delamination in cell wall. It is well known that the increment of both cumulative pore volume and specific surface area improve swelling, accessibility and the binding ability with matrix.

**References:**

1. Gharehkhani, S.; Sadeghinezhad, E.; Kazi, S.N.; Yarmand, H.; Badarudin, A.; Safaei, M.R.; Zubir, M.N.M. Basic effects of pulp refining on fiber properties—A review. CARBOHYD POLYM 2015, 115, 785-803. <https://doi.org/10.1016/j.carbpol.2014.08.047>
